# Characteristics of humanitarian aid in a developed country: Israel Defense Force’s experience from the Türkiye -Syria earthquake

**DOI:** 10.7189/jogh.13.03049

**Published:** 2023-08-25

**Authors:** Maya Nitecki, Sharon Ohayon, Ofer Almog

**Affiliations:** 1 Israel Defense Forces, Medical Corps, Ramat Gan, Israel; 2 Department of Military Medicine and “Tzameret”, Faculty of Medicine, Hebrew University of Jerusalem, Israel

On 6 February 2023, a series of powerful earthquakes hit southern Türkiye and northern Syria, causing massive destruction and affecting hundreds of thousands of lives. Over 45 000 people are reported to have been killed and more than 100 000 injured [1], with many more lives left in danger due to infrastructure collapse and freezing temperatures. In the immediate aftermath, efforts were focused on search, rescue, and providing medical care for entire communities that were left without fully functioning medical care facilities. The global relief response came in the form of medical supplies and personnel [2].

On 8 February, the Israel Defense Forces’ delegation, consisting of 140 members equipped with 17 tons of medical supplies and the capabilities of a level 3 field hospital [3], landed in Kahramanmaraş, a large city in southern Türkiye with a population over one million, consisting predominantly of Turkish Muslims and Kurds; however, due to its proximity to the Syrian border, the city hosts a relatively high number of Syrian refugees as well [4]. The IDFFH have had experience in relief missions throughout the world, operating as stand-alone, self-sufficient field hospitals [5-7], yet this mission was among the first to dispatch into an upper-middle-income country that previously had its own medical infrastructure. The plan was to operate a field hospital without support or utilities from the affected community, yet we soon realised that aid was required in a model we have never practised before.

The local Necip Fazıl Şehir Hastanesi public hospital that serves the local community, located only minutes away from massive destruction sites, had been left vacant, with most of the local staff and inpatients either being evacuated by Turkish authorities or having fled following the earthquakes. While the hospital building sustained minor structural damage, it had working electricity and running water, albeit with concern for water contamination. Prior to the disaster, the hospital provided ambulatory services which did not include paediatric, trauma, or gynaecological patient care. Realising the potential of this facility and following approval of its safety by engineers from the Israeli Search and Rescue team, our personnel re-opened the hospital and operated four departments: the emergency department (ED), a six-bed intensive care unit (ICU), the facility's five operating rooms and a 60-bed inpatient service. In one week, 470 patients received treatment, 150 of whom were children, including individuals rescued after being trapped over 100 hours under rubble. Additionally, the Israeli team performed 10 surgical procedures.

The dynamics of utilising a local facility in a developed country with organised central rehabilitation efforts had several unique characteristics. First, although we re-opened the hospital within 24 hours of our arrival and faced a growing influx of patients, the Turkish government also mobilised medical staff from across Türkiye to support the area, creating multi-cultural, multi-linguistic, and multi-disciplinary Turkish-Israeli teams. With the help of volunteer translators, these mixed teams delivered care in four languages (Turkish, English, Hebrew, and Arabic). The daily routine consisted of morning rounds, writing follow-up, admission, and discharge notes, and dividing tasks and shifts between all personnel. Documentation was carried out in two systems, the Israeli computerised electronic health record system (EHRS) and the Turkish partly computerised EHRS. Each working shift consisted of Israeli and Turkish personnel and one or two translators. Naturally, we encountered differences in medical approach and agreed to management through mutual decision. When a local or foreign had the ideal personnel for a specific case – for example, a paediatrician for a paediatric case or a neurosurgeon for a head trauma case – that team took up the managing of the patient.

The delivery of pharmacotherapy was particularly challenging and required careful planning. As both teams were unfamiliar with the facility pharmacy supply or the other team's supply, administration of medication often took longer than anticipated. After 48 hours of operation, we identified the need for training inside pharmacy rooms and around crash carts, which we held with the arrival of any new team member. The responsibility for additional testing such as laboratory work, imaging, and pharmacy services was based on local teams and the hospital’s infrastructure, with the Israeli team being responsible for laboratories and medications not routinely available at the facility, such as amiodarone or digoxin. Relying on local infrastructure for laboratories and imaging allowed for direct integration of results in the Turkish EHRS.

An advantage of working inside a local facility was the ability to admit patients into an already existing large inpatient service and discharge them with the guidance from local caregivers familiar with ambulatory services available in the community. This was crucial in supporting high bed turnover in the ICU, which ultimately allowed for admission and care for more patients.

This collaborative and integrative model, previously described by Merin et al. [7], allowed us to care for patients in a cultural and physical setting familiar to them and through the infrastructures they are accustomed to, respecting their right not only to health, but health given in reasonable timing and distance from their place of residence.. This principle is important in a country and community rehabilitating after a disaster, yet it is only feasible when local facilities are partially functional. This model of integrative work with a local team was also important in gaining the trust of the local community in a complex political reality.

Finally, leaving a disaster area can create ethical dilemmas such as leaving a community without sufficient medical care and without the assurance of its continuity. Yet, in this model of cooperation inside the local infrastructure in a developed country, in a span of merely a week, the local hospital returned almost fully to its pre-disaster capacity, re-opening a second inpatient floor, staffing the entire ER department, and even treating patients it would not normally treat, such as trauma and paediatric patients. This allowed our humanitarian aid delegation to leave, after passing the baton to the Turkish teams, with the assurance of continuity of care, hopefully ensuring the success and sustainability of aid efforts.

We believe that our unique experience has taught us valuable lessons that could be beneficial for future relief missions operating in a collaborative model. Working as part of a multi-cultural and multi-disciplinary team can be challenging due to varying treatment approaches and practice standards. Some of the differences we encountered included timing of catheter removal, type and rate of fluid administration, antibiotic administration, and pain management protocols. In our view, it was essential to respect the practices and decisions of the local teams. The purpose of any humanitarian aid should be to help people in any way necessary, rather than impose a certain standard of care on the local health system. Other necessary traits in this context were flexibility and adaptability. One should be prepared and willing to assume different roles within the team, as needs might change rapidly, keeping in mind that help may be needed in unexpected ways. Finally, patience is crucial for successful communication in this setting. Building relationships and trust takes time, and investing time to reinforce communication through translators is essential in establishing them.

When local infrastructure is at least partly operational after a disaster, an encounter often seen in high- and middle-income countries, a collaborative model with local relief teams may have several advantages to local disaster victims, allowing for swift administration of aid and, in a relatively short period, facilitating the restoration of local health facilities.
